# Advanced Constitutive Modeling of the Hot Deformation Behavior of Ni-Based Superalloys: Modified Kobayashi–Dodd and Khan–Huang–Liang Models with Experimental Validation

**DOI:** 10.3390/ma18112500

**Published:** 2025-05-26

**Authors:** Ali Abd El-Aty, Yong Xu, Bandar Alzahrani, Alamry Ali, Abdallah Shokry

**Affiliations:** 1 Department of Mechanical Engineering, College of Engineering at Al Kharj, Prince Sattam Bin Abdulaziz University, Al Kharj 11942, Saudi Arabia; 2 Institute of Metal Research, Chinese Academy of Sciences, Shenyang 110016, China; 3 Department of Mechanical Engineering, Faculty of Engineering, Fayoum University, Fayoum 63514, Egypt

**Keywords:** sustainability, superalloy, constitutive model, hot deformation, Kobayashi–Dodd, Khan–Huang–Liang, SDGs

## Abstract

The hot deformation behavior of nickel-based superalloy 925 under different strain rates and elevated temperatures is inherently complex because of its strong dependence on strain, strain rate, and temperature. Constitutive modeling becomes essential to capture and predict this behavior accurately. In this study, two modified versions of the Kobayashi–Dodd (KD) and the Khan–Huang–Liang (KHL) models were introduced based on a detailed analysis of the alloy’s hot deformation characteristics, aiming to increase their predictive capabilities. The accuracy of the modified models, along with their original versions, was rigorously evaluated via the key statistical parameters correlation coefficient (R), average absolute relative error (AARE), and root mean square error (RMSE). The findings revealed that the modified KD and KHL models agreed well with the experimental data, indicating a remarkable fit. Both modified versions demonstrated high predictive accuracy, each achieving an R-value of 0.997. The modified KHL model reported an AARE of 3.07% and an RMSE of 7.49 MPa, whereas the modified KD model achieved an AARE of 3.19% and an RMSE of 6.64 MPa. These results confirm that the improved models offer superior performance in capturing the hot flow behavior of nickel-based superalloy 925, making them valuable tools for high-temperature forming process simulations and improving the overall performance of components. Furthermore, by enabling more efficient and optimized forming processes, these models contribute to sustainable manufacturing by minimizing material waste and optimizing energy usage, supporting global sustainability initiatives in line with SDG 9, SDG 12, and SDG 13.

## 1. Introduction

Nickel-based superalloy 925 is a high-strength, corrosion-resistant material engineered for exceptional performance in extreme environments, particularly at elevated temperatures [1]. Due to its excellent properties, Superalloy 925 is extensively employed in demanding sectors, such as aerospace, oil and gas, and marine applications [2]. However, the response of alloys to hot deformation is highly complex and influenced by key factors, including temperature, strain rate, and microstructural evolution [3,4,5]. A thorough understanding of these variables is essential for optimizing manufacturing processes and ensuring that an alloy maintains its structural integrity under service conditions [6]. In particular, when subjected to harsh, high-temperature environments, the flow behavior of alloy 925 becomes critical, presenting challenges that necessitate detailed thermomechanical analysis [7].

Although the primary production of Ni-based superalloys, such as alloy 925, is inherently energy intensive and relies on critical raw materials, such as cobalt and rhenium, recent advancements in processing strategies offer significant potential for improving sustainability [8]. Optimized hot deformation techniques and process-integrated modeling approaches can reduce energy consumption by up to 30% and material waste by 20–40% during the manufacturing of high-performance components [9]. These challenges are not solely technical but are increasingly interlinked with broader sustainability imperatives [10]. The environmental and ethical concerns associated with resource extraction are counterbalanced by the long service life, recyclability, and energy-efficiency benefits that Ni-based superalloys provide in demanding applications [11]. As such, developing a deeper understanding of the hot deformation behavior of alloy 925 is essential not only for performance optimization but also for advancing sustainable manufacturing practices [12]. This effort directly supports several United Nations Sustainable Development Goals (SDGs), particularly SDG 9 (Industry, Innovation, and Infrastructure), SDG 12 (Responsible Consumption and Production), and SDG 13 (Climate Action) [12,13]. Thus, research on alloy 925 represents a strategic intersection between advanced materials engineering and global sustainability objectives.

A crucial pathway for enhancing the sustainability of Ni-based superalloys lies in the application of constitutive modeling for hot deformation behavior [13]. Whether empirical or physically based, these models effectively characterize how such alloys respond to varying stress, strain rates, and temperature conditions [14]. This capability allows the design and optimization of manufacturing processes that minimize energy consumption, material waste, and production time [15]. As a result, constitutive modeling plays a pivotal role in advancing cleaner, more resource-efficient industrial practices [16]. Moreover, when combined with finite element simulations and machine learning, these models support innovative, adaptive manufacturing approaches that align with broader sustainability targets, particularly SDG 7 (Affordable and Clean Energy) and SDG 17 (Partnerships for the Goals), by promoting resource conservation and data-driven innovation [17].

In this context, modeling the hot flow behavior of nickel-based superalloy 925 is particularly critical. As a material extensively used in high-temperature applications, accurate deformation characterization is essential to prevent defects, ensure dimensional accuracy, and maintain structural integrity during manufacturing [18]. Constitutive models help establish precise mathematical relationships between stress, strain, strain rate, and temperature, providing engineers with the tools to design efficient and reliable processes such as forging, extrusion, and rolling [18,19]. These models reduce the reliance on costly trial-and-error methods and enhance the predictive power of numerical simulations, enabling better control over material behavior and process outcomes [20]. Integrating constitutive modeling for alloy 925 ultimately reinforces sustainable manufacturing goals while maintaining the high-performance standards required in critical industrial applications [18,19,20].

Constitutive modeling of hot deformation can be approached through physical, phenomenological, and machine learning-based models [21,22]. Physical models are grounded in fundamental principles of deformation mechanisms, incorporating microstructural evolution, such as dislocation dynamics, recovery, and recrystallization [23,24,25]. Phenomenological models rely on empirical relationships to describe the flow stress based on experimental data, making them widely used for practical applications [26,27,28]. Moreover, machine learning-based models leverage advanced computational techniques, such as neural networks and genetic algorithms, to accurately predict material behavior by analyzing complex, nonlinear relationships within large datasets [29,30,31]. Several modified constitutive models have been developed to increase the accuracy of predicting the hot flow behavior of various element-based alloys, incorporating both phenomenological [32,33,34,35,36,37,38] and physically based [39,40,41,42,43,44] approaches.

In 1989, Kobayashi and Dodd (KD) [45] developed a phenomenological model to describe the hot deformation behavior of metals and alloys across different temperatures and strain rates. The KD model independently considers strain hardening, dynamic recovery, and softening, providing a reliable framework for analyzing material flow at elevated temperatures. One of its notable advantages is its simplicity, as it requires only four constants for accurate characterization. Similarly, Khan Huang and Liang (KHL) [46] introduced another widely adopted phenomenological model for predicting the flow behavior of metals and alloys during hot deformation. The KHL model effectively accounts for the effects of strain, strain rate, and temperature on flow stress, making it a valuable tool for high-temperature forming processes. Owing to the nonlinear and complex nature of material behavior, both the KD and KHL models sometimes struggle to accurately predict flow behavior at elevated temperatures and varying strain rates [47,48,49,50,51,52,53]. As a result, advanced modeling techniques and modifications to existing KD and KHL models are crucial for improving prediction accuracy. Enhancing these models allows for a more precise representation of material deformation, optimizing hot working conditions and better replicating real-world applications. This, in turn, contributes to extending the service life of components and improving overall performance.

This study introduces two advanced modified versions of the KD and KHL models to increase the accuracy of predicting the hot flow behavior of nickel-based superalloy 925. The original KD and KHL models and their modified versions are analyzed and evaluated via the performance metrics R, AARE, and RMSE. The material parameters are identified through a nonlinear regression approach, which uses the Levenberg–Marquardt algorithm to minimize the sum of the squared errors.

## 2. Experimentation

Zhu et al. [54] investigated the hot deformation behavior of nickel-based superalloy 925. Their study examined the alloy under strain rates of 0.01, 0.1, 1, and 10 s^−1^ and temperatures ranging from 900 °C to 1150 °C, specifically 900, 950, 1000, 1050, 1100, and 1150 °C. Hot tensile tests were conducted via a Gleeble-3500 thermomechanical simulator. Details on the chemical preparation of the samples, composition, microstructure, and stress–strain curves at various strain rates and temperature combinations can be found in [54].

In the initial stage of hot deformation, the flow stress increases rapidly due to the predominance of strain hardening. As the strain rate increases, the formation and multiplication of dislocations become the dominant mechanism, further increasing the flow stress. Conversely, as the temperature increases, a reduction in flow stress is observed, primarily due to softening effects. During this phase, dynamic recrystallization occurs relatively slowly, contributing to the gradual decline in flow stress.

In this study, stress and strain values are obtained from the stress–strain data published in [54] via the open-source software Plot Digitizer 2.6.11b. The two modified versions, MKD and MKHL, and the original KD and KHL models are developed and analyzed. A comparative assessment was conducted to determine the most suitable model for accurately predicting the flow behavior of nickel-based superalloy 925 under various temperatures and strain rates. Although an independent hold-out dataset was unavailable, potential overfitting was addressed through two key measures. First, a comprehensive and dense dataset comprising approximately 320 data points across 16 stress–strain curves covering 4 distinct strain rates and 4 temperature levels was employed, ensuring robust coverage of the deformation domain. Second, the predicted model responses were systematically validated against well-established trends reported in the literature for nickel-based superalloys. These include the characteristic increase in flow stress with increasing strain rate, attributed to dislocation-dominated mechanisms, and the decrease in flow stress with elevated temperatures, typically associated with dynamic recrystallization and thermal softening phenomena [55,56,57,58].

## 3. Constitutive Modeling

Constitutive models used to predict the hot deformation behavior of metallic alloys serve as mathematical tools that describe the relationships among stress, strain, temperature, and strain rate during high-temperature manufacturing processes, such as forging, rolling, and extrusion. These models incorporate material-specific parameters, such as activation energy, flow stress, and microstructural evolution to account for key deformation mechanisms, including dislocation motion, grain boundary sliding, and phase transformations. The development of accurate constitutive models is essential for optimizing processing conditions and enhancing the mechanical performance and structural integrity of materials in elevated-temperature applications.

This section presents detailed modifications of the Kobayashi–Dodd model (KD) and the Khan–Huang–Liang model (KHL) to improve the predictive capabilities of these models. The constants are estimated via MATLAB, which employs an iterative process that begins with the suggested initial values. This process fits a nonlinear regression model by minimizing the sum of squared errors through the Levenberg–Marquardt algorithm [59]. In this procedure, experimental stress is treated as the response variable, whereas strain, strain rate, and temperature are predictor variables. The Levenberg–Marquardt algorithm is described by [59]:

(1) $$x_{k+1}=x_{k}-{\left[J^{T}J+\mu I\right]}^{-1}J^{T}\left(z-h\left(x_{k}\right)\right)$$

where x is a vector that contains the model constants, represented as $x={\left[x_{1},\;{\;x}_{2},\;\ldots ,\;x_{t}\right]}^{T}$, with t indicating the total number of constants. When parameter μ is set to zero, the Levenberg–Marquardt algorithm reduces to the Gauss–Newton algorithm, where μ represents a scalar value, and I is the identity matrix. Parameter z represents a vector comprising experimental stress values expressed as ${\left[z_{1},\;{\;z}_{2},\;\ldots ,\;z_{N}\right]}^{T}$, where N indicates the total number of stress measurements. Parameter $h\left(x_{k}\right)$ represents a vector of predicted stress at $x_{k}$. Parameter J refers to a Jacobian matrix of size N×t, which contains the derivatives of $h\left(x_{k}\right)$ with respect to $x_{k}$. The matrix J is given by:

(2) $$J=\left[\begin{array}{ccc} \frac{{\partial h}_{1}}{{\partial x}_{1}} & \ldots & \frac{{\partial h}_{1}}{{\partial x}_{t}}\\ \vdots & \ddots & \vdots \\ \frac{{\partial h}_{N}}{{\partial x}_{1}} & \ldots & \frac{{\partial h}_{N}}{{\partial x}_{t}} \end{array}\right]$$

### 3.1. Kobayashi–Dodd Model (KD)

In 1989, Kobayashi and Dodd [45] introduced a phenomenological model to predict the hot deformation behavior of metals and alloys under various temperatures and strain rates. In the KD model, strain hardening, dynamic recovery, and softening are coupled independently. The KD model is introduced as follows:

(3) $$\sigma =\sigma_{0}\;\varepsilon^{n}\;{\varepsilon^{\cdot }}^{m}\left(1-\beta ∆T\right)$$

where σ represents the flow stress, ε denotes the plastic strain, and $\varepsilon^{\cdot }$ represents the strain rate. $∆T=T-T_{r}$ defines the difference in temperature between the testing temperature, T, and a selected reference temperature, $T_{r}$, while $\sigma_{0},\;n,\;m,$ and β represent material constants.

Using initial values of 140, 0.1, −0.1, and 0.02 for $x_{0}$, the Levenberg–Marquardt algorithm successfully converges to the optimal parameters that minimize the sum of the squared errors between the experimental and predicted stress from the KD model. Table 1 contains the determined constants of the KD model for superalloy 925. The final KD model can be presented as:

(4) $$\sigma =259.86\;\varepsilon^{-0.081}\;{\varepsilon^{\cdot }}^{\left(0.143\right)}\left(1-0.0032\;∆T\right)$$

### 3.2. Khan–Huang–Liang Model (KHL)

The KHL model [46] is a widely recognized phenomenological model used to predict the flow behavior of metals and alloys during hot deformation. The KHL model is introduced as follows:

(5) $$\sigma =\left[A+B{\left(1-\frac{\ln\varepsilon^{\cdot }}{\ln D_{0}^{p}}\right)}^{n_{1}}\varepsilon^{n_{0}}\right]e^{C\ln\varepsilon^{\cdot }\;}\left(1-{T^{*}}^{m}\right)$$

In this context, σ, ε, and $\varepsilon^{\cdot }$ represent the stress, strain, and strain rates, respectively. The term $T^{*}$ denotes the ratio of $T-T_{r}$ to $T_{m}-T_{r}$, where T is the tested temperature, $T_{r}$ is the reference temperature, and $T_{m}$ is the melting temperature. The material constants include A, B, $n_{1}$, $n_{0}$, C, and m, whereas $D_{0}^{p}$ is an arbitrarily selected strain rate value set to 100 s^−1^ for this study.

At a chosen reference strain rate of 1 s^−1^ and a chosen temperature of 1000 °C, A is the yield stress, which is determined to be 316.5 MPa. With initial values of 55, 0.7, −0.35, 0.25, and 0.6 for $x_{0}$, the Levenberg–Marquardt algorithm effectively converges to the optimal parameters that minimize the sum of the squared errors between the experimental stress and the predicted stress from the KHL model. Table 2 contains the determined constants of the KHL model for superalloy 925. The resulting KHL model can be expressed as follows:

(6) $$\sigma =\left[316.50-25.64{\left(1-\frac{\ln\varepsilon^{\cdot }}{\ln D_{0}^{p}}\right)}^{2.899}\varepsilon^{0.322}\right]e^{0.075\;\ln\varepsilon^{\cdot }\;}\left(1-{T^{*}}^{0.933}\right)$$

### 3.3. Modified Kobayashi–Dodd Model (MKD)

At a reference strain rate of 1 s^−1^ and a reference temperature of 1000 °C, while considering the strain hardening exponent as a function of ε, Equation (3) simplifies to:

(7) $$\sigma =\sigma_{0}\;\varepsilon^{n\left(\varepsilon \right)}$$

**Figure 1:**
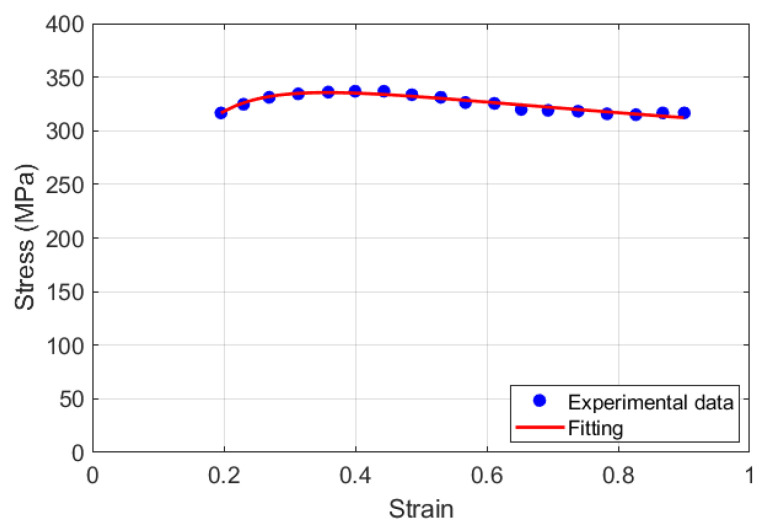
Stress versus strain at reference conditions for the KD model.

By fitting the experimental stress–strain data under reference conditions (cf. Figure 1), *n*(*ε*) can be determined as follows:

(8) $$\sigma =307.85\;\varepsilon^{\left(-0.165+0.029/\varepsilon \right)}$$

Therefore, a general form for Equation (8) can be introduced as:

(9) $$\sigma =\sigma_{0}\;\varepsilon^{\left(n_{0}+n_{1}/\varepsilon \right)}$$

At the chosen reference temperature and considering Equation (7), Equation (3) can be written as:

(10) $$\sigma =\sigma_{0}\;\varepsilon^{\left(n_{0}+n_{1}/\varepsilon \right)}\;{\varepsilon^{\cdot }}^{m}$$

By taking the logarithm and performing some rearrangements, with the strain rate parameter m treated as a function of both ε and $\varepsilon^{\cdot }$, m can be expressed as follows:

(11) $$m\left(\varepsilon ,\varepsilon^{\cdot }\right)=\frac{\ln\left(\sigma /\sigma_{0}\;\varepsilon^{\left(n_{0}+n_{1}/\varepsilon \right)}\;\right)}{\ln\varepsilon^{\cdot }}$$

**Figure 2:**
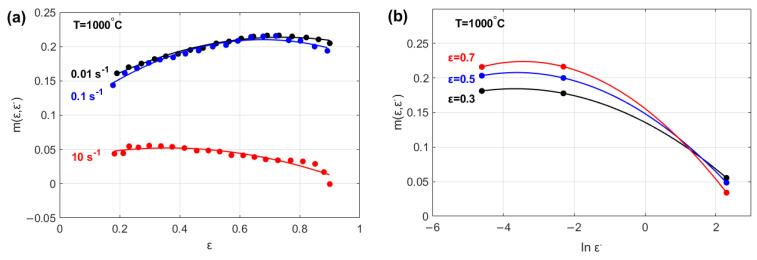
Strain rate sensitivity exponent, $m\;\left(\varepsilon ,\varepsilon^{\cdot }\right)$ vs. (**a**) strain, ε, and (**b**) strain rate, $\varepsilon^{\cdot }$.

The effects of both ε and $\varepsilon^{\cdot }$ on parameter m are shown in Figure 2. The figure shows that parameter $m\left(\varepsilon ,\varepsilon^{\cdot }\right)$ can be modeled as a quadratic function of both the strain (Figure 2a) and strain rate (Figure 2b).

Therefore, the $m\left(\varepsilon ,\varepsilon^{\cdot }\right)$ parameter can be proposed as:

(12) $$m\left(\varepsilon ,\varepsilon^{\cdot }\right)=m_{0}+m_{1}\varepsilon +m_{2}\varepsilon^{\cdot }+m_{3}\varepsilon^{2}{\varepsilon^{\cdot }}^{2}$$

The initial values for constants $m_{0}$, $m_{1}$, $m_{2}$, and $m_{3}$ are chosen as 0.156, 0.075, −0.012, and −0.0012, respectively.

Considering Equations (10) and (12), after taking the logarithm and rearranging Equation (3), parameter $\beta \left(\varepsilon ,\;\varepsilon^{\cdot },\;∆T\right)$ can be expressed as a function of ε, $\varepsilon^{\cdot }$, and ∆T, which accounts for the softening effect:

(13) $$\beta \left(\varepsilon ,\;\varepsilon^{\cdot },\;∆T\right)=\left[1-\frac{\sigma }{\sigma_{0}\varepsilon^{n\left(\varepsilon \right)}{\varepsilon^{\cdot }}^{m\left(\varepsilon ,\varepsilon^{\cdot }\right)}}\right]/∆T$$

**Figure 3:**
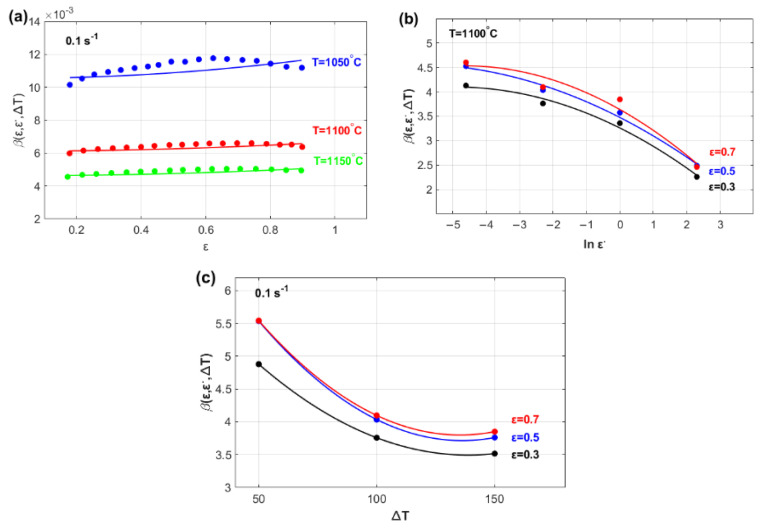
Temperature sensitivity parameter, $\beta \left(\varepsilon ,\;\varepsilon^{\cdot },\;∆T\right)$ vs. (**a**) strain, ε; (**b**) strain rate, $\varepsilon^{\cdot }$; and (**c**) temperature, ∆T.

The effects of ε, $\varepsilon^{\cdot }$, and ∆T on parameter β are shown in Figure 3. The figure shows that parameter $\beta \left(\varepsilon ,\;\varepsilon^{\cdot },\;∆T\right)$ can be modeled as a linear function of strain (Figure 3a) and quadratic functions of both the strain rate (Figure 3b) and the temperature (Figure 3c).

As a result, parameter $\beta \left(\varepsilon ,\;\varepsilon^{\cdot },\;∆T\right)$ can be proposed as follows:

(14) $$\beta \left(\varepsilon ,\;\varepsilon^{\cdot },\;∆T\right)=\beta_{0}+\beta_{1}\varepsilon +\beta_{2}\varepsilon^{\cdot }+\beta_{3}∆T+\beta_{4}{\varepsilon^{\cdot }}^{2}{∆T}^{2}$$

The initial values for constants $\beta_{0}$, $\beta_{1}$, $\beta_{2}$, $\beta_{3}$, and $\beta_{4}$ are 0.0045, 0.0004, −0.0002, −7.25 × 10^−6^, and 5.6 × 10^−10^, respectively.

The MKD model can now be expressed in the following form:

(15) $$\sigma =\sigma_{0}\;\varepsilon^{n\left(\varepsilon \right)}\;{\varepsilon^{\cdot }}^{m\left(\varepsilon ,\varepsilon^{\cdot }\right)}\left(1-\beta \left(\varepsilon ,\;\varepsilon^{\cdot },\;∆T\right)∆T\right)$$

(16) $$n\left(\varepsilon \right)=n_{0}+\frac{n_{1}}{\varepsilon }$$

(17) $$m\left(\varepsilon ,\varepsilon^{\cdot }\right)=m_{0}+m_{1}\varepsilon +m_{2}\varepsilon^{\cdot }+m_{3}\varepsilon^{2}{\varepsilon^{\cdot }}^{2}$$

(18) $$\beta \left(\varepsilon ,\;\varepsilon^{\cdot },\;∆T\right)=\beta_{0}+\beta_{1}\varepsilon +\beta_{2}\varepsilon^{\cdot }+\beta_{3}∆T+\beta_{4}{\varepsilon^{\cdot }}^{2}{∆T}^{2}$$

By using the initial values obtained for constants $n_{0}$, $n_{1}$, $m_{0}$, $m_{1}$, $m_{2}$, $m_{3}$, $\beta_{0}$, $\beta_{1}$, $\beta_{2}$, $\beta_{3}$, and $\beta_{4}$ in the Levenberg–Marquardt algorithm, the final constants can be derived. Table 3 contains the determined constants of the MKD model for superalloy 925. The MKD model, along with these constants, can then be presented as follows:

(19) $$\sigma =311.61\;\varepsilon^{n\left(\varepsilon \right)}\;{\varepsilon^{\cdot }}^{m\left(\varepsilon ,\varepsilon^{\cdot }\right)}\left(1-\beta \left(\varepsilon ,\;\varepsilon^{\cdot },\;∆T\right)∆T\right)$$

(20) $$n\left(\varepsilon \right)=-0.137+\frac{0.018}{\varepsilon }$$

(21) $$m\left(\varepsilon ,\varepsilon^{\cdot }\right)=0.171+0.072\varepsilon -0.015\varepsilon^{\cdot }-0.0009\varepsilon^{2}{\varepsilon^{\cdot }}^{2}$$

(22) $$\beta \left(\varepsilon ,\;\varepsilon^{\cdot },\;∆T\right)=0.0045+0.0003\varepsilon -0.0002\varepsilon^{\cdot }-7.19\times 10^{-06}∆T+5.94\times 10^{-10}{\varepsilon^{\cdot }}^{2}{∆T}^{2}$$

### 3.4. Modified Khan–Huang–Liang Model (MKHL)

At a reference strain rate of 1 s^−1^ and a reference temperature of 1000 °C, Equation (5) reduces to:

(23) $$\sigma =A+B\varepsilon^{n_{0}}$$

**Figure 4:**
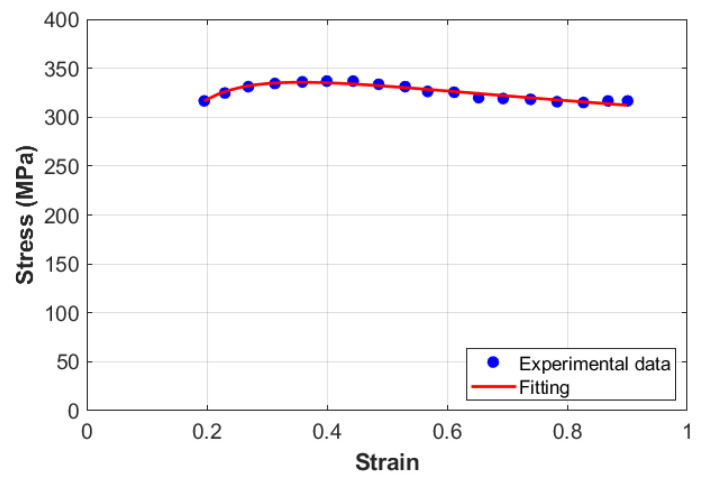
Stress versus strain at reference conditions for the KHL model.

Constant A is given by the yield stress at reference conditions, which is 316.50 MPa, whereas the two constants *B* and $n_{0}$ are determined from fitting as 5.925 MPa and −0.545, respectively, which are used later as initial values for *B* and $n_{0}$ (cf. Figure 4).

At the reference temperature, Equation (5) reduces to:

(24) $$\sigma =\left[A+B{\left(1-\frac{\ln\varepsilon^{\cdot }}{\ln D_{0}^{p}}\right)}^{n_{1}}\varepsilon^{n_{0}}\right]e^{C\ln\varepsilon^{\cdot }\;}$$

Neglecting the effect of the strain rate inside the strain hardening term, i.e., $n_{1}=0$, parameter C can be expressed as a function of ε and $\varepsilon^{\cdot }$, in which Equation (24) can be rewritten as:

(25) $$C\left(\varepsilon ,\varepsilon^{\cdot }\right)=\frac{\ln\left[\sigma /\left(A+B\varepsilon^{n_{0}}\right)\right]}{\ln\varepsilon^{\cdot }}$$

**Figure 5:**
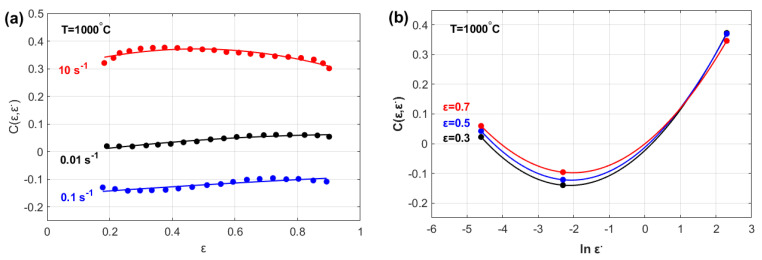
Strain rate sensitivity parameter, $C\left(\varepsilon ,\varepsilon^{\cdot }\right)$ vs. (**a**) strain, ε, and (**b**) strain rate, $\varepsilon^{\cdot }$.

The effects of ε and $\varepsilon^{\cdot }$ on parameter C are illustrated in Figure 5. As shown, parameter C can be represented as a quadratic function of both ε (Figure 5a) and $\varepsilon^{\cdot }$ (Figure 5b).

Accordingly, both strain rate parameters C and $n_{1}$ can be proposed as follows:

(26) $$C\left(\varepsilon ,\varepsilon^{\cdot }\right)=C_{0}+C_{1}\varepsilon +C_{2}\varepsilon^{\cdot }+C_{3}\varepsilon^{2}{\varepsilon^{\cdot }}^{2}$$

(27) $$n_{1}\left(\varepsilon ,\varepsilon^{\cdot }\right)=n_{10}+n_{11}\varepsilon +n_{12}\varepsilon^{\cdot }+n_{13}\varepsilon^{2}{\varepsilon^{\cdot }}^{2}$$

At the reference temperature, the initial values for constants $C_{0}$, $C_{1}$, $C_{2}$, $C_{3}$, $n_{10}$, $n_{11}$, $n_{12}$, and $n_{13}$ are 0.422, 0.234, −0.173, −0.054, 9.942, 4.336, −0.249, and −0.451, respectively.

After taking the logarithm and rearranging Equation (5), parameter $m\left(\varepsilon ,\;\varepsilon^{\cdot },\;∆T\right)$ can be expressed as a function of ε, $\varepsilon^{\cdot }$, and ∆T, which captures the softening effect as:

(28) $$m\left(\varepsilon ,\;\varepsilon^{\cdot },\;∆T\right)=\ln\left[1-\frac{\sigma }{\left[A+B{\left(1-\frac{\ln\varepsilon^{\cdot }}{\ln D_{0}^{p}}\right)}^{n_{1}\left(\varepsilon ,\;\varepsilon^{\cdot }\right)}\varepsilon^{n_{0}}\right]e^{C\left(\varepsilon ,\;\varepsilon^{\cdot }\right)\ln\varepsilon^{\cdot }\;}}\right]/\ln T^{*}$$

**Figure 6:**
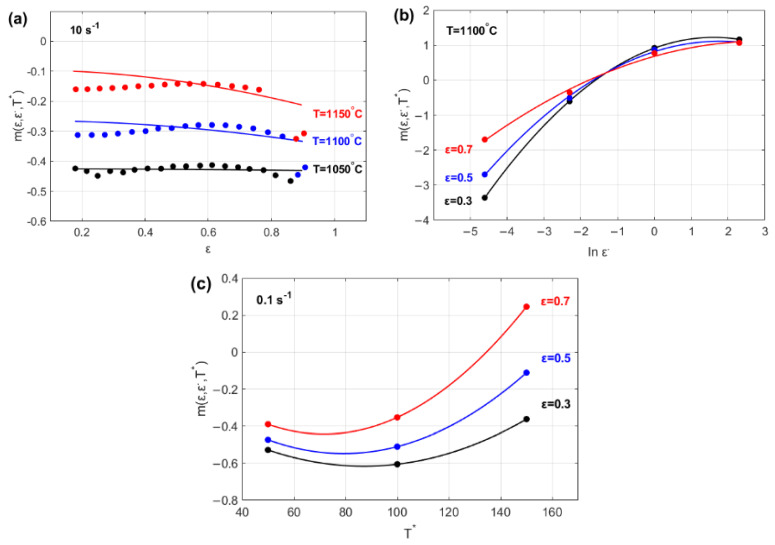
Temperature sensitivity parameter, $m\left(\varepsilon ,\;\varepsilon^{\cdot },\;∆T\right)$, versus (**a**) strain, ε; (**b**) strain rate, $\varepsilon^{\cdot }$; and (**c**) temperature, $T^{*}$.

The effects of *ε*, $\varepsilon^{\cdot }$ and ∆*T* on parameter m are illustrated in Figure 6. The figure indicates that parameter *m*(*ε*, $\varepsilon^{\cdot }$, ∆*T*) might be modeled as a linear function of strain (Figure 6a) and as quadratic functions of both the strain rate (Figure 6b) and temperature (Figure 6c).

Consequently, parameter $m\left(\varepsilon ,\;\varepsilon^{\cdot },\;∆T\right)$ can be proposed as follows:

(29) $$m\left(\varepsilon ,\;\varepsilon^{\cdot },\;∆T\right)=m_{0}+m_{1}\varepsilon +m_{2}\varepsilon^{\cdot }+m_{3}T^{*}+m_{4}{\varepsilon^{\cdot }}^{2}{T^{*}}^{2}$$

The initial values for constants $m_{0}$, $m_{1}$, $m_{2}$, $m_{3}$, and $m_{4}$ are 0.988, −0.145, 0.059, −0.150, and 0.041, respectively.

The final MKHL model can now be expressed in the following form:

(30) $$\sigma =\left[A+B{\left(1-\frac{\ln\varepsilon^{\cdot }}{\ln D_{0}^{p}}\right)}^{n_{1}\left(\varepsilon ,\;\varepsilon^{\cdot }\right)}\varepsilon^{n_{0}}\right]e^{C\left(\varepsilon ,\;\varepsilon^{\cdot }\right)\ln\varepsilon^{\cdot }\;}\left(1-{T^{*}}^{m\left(\varepsilon ,\;\varepsilon^{\cdot },\;T^{*}\right)}\right)$$

(31) $$n_{1}\left(\varepsilon ,\;\varepsilon^{\cdot }\right)=n_{10}+n_{11}\varepsilon +n_{12}\varepsilon^{\cdot }+n_{13}\varepsilon^{2}\varepsilon^{\cdot 2}$$

(32) $$C\left(\varepsilon ,\;\varepsilon^{\cdot }\right)=C_{0}+C_{1}\varepsilon +C_{2}\varepsilon^{\cdot }+C_{3}\varepsilon^{2}\varepsilon^{\cdot 2}$$

(33) $$m\left(\varepsilon ,\;\varepsilon^{\cdot },\;∆T\right)=m_{0}+m_{1}\varepsilon +m_{2}\varepsilon^{\cdot }+m_{3}T^{*}+m_{4}{\varepsilon^{\cdot }}^{2}{T^{*}}^{2}$$

By using the initial values chosen for constants $n_{10}$, $n_{11}$, $n_{12}$, $n_{13}$, $C_{0}$, $C_{1}$, $C_{2}$, $C_{3}$, $m_{0}$, $m_{1}$, $m_{2}$, $m_{3}$, and $m_{4}$ in the Levenberg–Marquardt algorithm, the final constants can be derived. Table 4 contains the determined constants of the MKHL model for superalloy 925. The MKHL model, incorporating these constants, can then be presented as follows:

(34) $$\sigma =\left[316.50+6.54{\left(1-\frac{\ln\varepsilon^{\cdot }}{\ln D_{0}^{p}}\right)}^{n_{1}\left(\varepsilon ,\;\varepsilon^{\cdot }\right)}\varepsilon^{-0.045}\right]e^{C\left(\varepsilon ,\;\varepsilon^{\cdot }\right)\ln\varepsilon^{\cdot }\;}\left(1-{T^{*}}^{m\left(\varepsilon ,\;\varepsilon^{\cdot },\;T^{*}\right)}\right)$$

(35) $$n_{1}\left(\varepsilon ,\;\varepsilon^{\cdot }\right)=12.004+0.417\varepsilon +0.225\varepsilon^{\cdot }-0.244\varepsilon^{2}{\varepsilon^{\cdot }}^{2}$$

(36) $$C\left(\varepsilon ,\;\varepsilon^{\cdot }\right)=0.349+0.140\varepsilon -0.134\varepsilon^{\cdot }-0.035\varepsilon^{2}{\varepsilon^{\cdot }}^{2}$$

(37) $$m\left(\varepsilon ,\;\varepsilon^{\cdot },\;∆T\right)=1.023-0.198\varepsilon +0.063\varepsilon^{\cdot }-0.141T^{*}+0.032\varepsilon^{\cdot 2}{T^{*}}^{2}$$

## 4. Results and Discussion

### 4.1. Comparison of the Predicted and Experimental Stress

**Figure 7:**
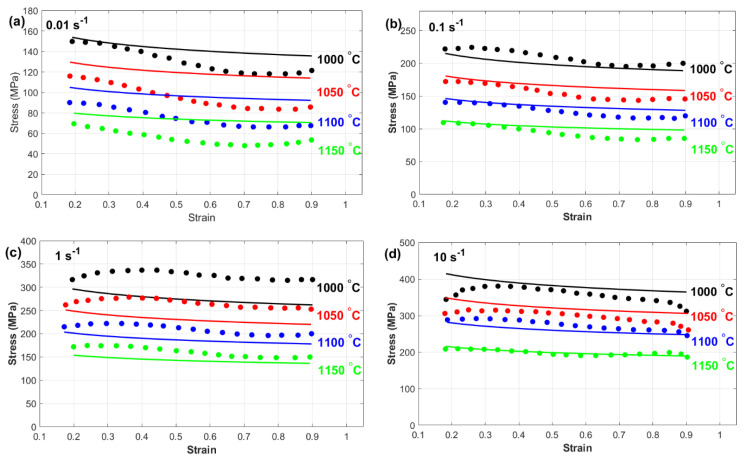
Experimental stress (**markers**) versus predicted stress obtained by the KD model (**lines**) at (**a**) 0.01 s^−1^, (**b**) 0.1 s^−1^, (**c**) 1 s^−1^, and (**d**) 10 s^−1^.

This subsection provides a comprehensive comparison between the experimentally measured stress and the predicted stress obtained from the Kobayashi–Dodd (KD) and Khan–Huang–Liang (KHL) models, along with their modified versions (MKD and MKHL). The accuracy and reliability of these models in capturing the deformation behavior of nickel-based Superalloy 925 were examined in detail. Furthermore, the discussion highlights the strengths and limitations of each model, emphasizing the improvements achieved through the modified formulations. Figure 7 shows a comparison between the experimentally measured stress and those predicted by the Kobayashi–Dodd (KD) model. The results demonstrate that the KD model struggles to accurately capture the flow behavior across different strain rates and temperature conditions, as evident in Figure 7a–d. This discrepancy suggests that the model does not fully account for the complex interactions between key deformation parameters.

One possible reason for this deviation is the lack of proper correlation between strain, strain rate, and temperature within the KD model formulation. Since hot deformation is a highly nonlinear process influenced by dynamic microstructural changes, the inability to incorporate these interdependencies can lead to significant errors in stress prediction. Specifically, at higher temperatures and strain rates, where dynamic recovery and recrystallization play crucial roles, the KD model’s limitations become more pronounced. As a result, the KD model may not be suitable for precise flow stress prediction, particularly for materials, such as nickel-based superalloy 925, which exhibits complex deformation mechanisms. This highlights the necessity for modifications that integrate strain, strain rate, and temperature effects more effectively to increase the predictive accuracy.

**Figure 8:**
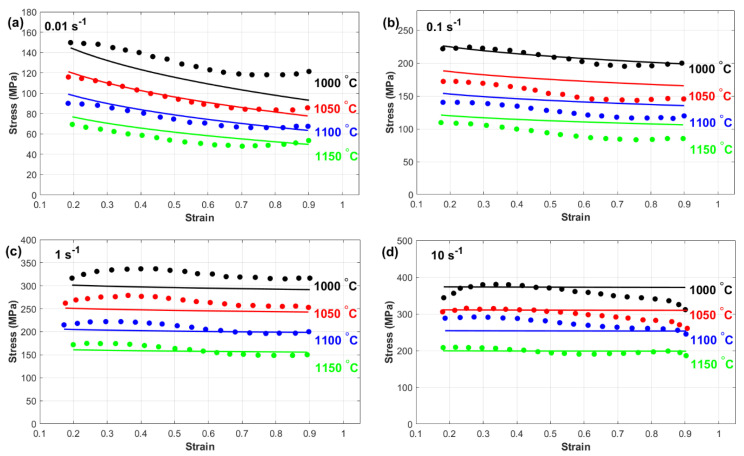
Experimental stress (**markers**) versus predicted stress obtained by the KHL model (**lines**) at (**a**) 0.01 s^−1^, (**b**) 0.1 s^−1^, (**c**) 1 s^−1^, and (**d**) 10 s^−1^.

Figure 8 presents a comparison between the experimentally measured stress and the predicted stress obtained from the Khan–Huang–Liang (KHL) model. The results indicate that in many cases (Figure 8a,b, and many combinations at Figure 8c,d), the KHL model does not fully capture the hot flow behavior of the tested alloy across a wide range of strain rates and temperatures. This inconsistency suggests that the model has limitations in accurately representing the material’s response under varying thermomechanical conditions. One potential reason for this discrepancy is the inherent complexity of the alloy’s flow behavior during hot deformation. The KHL model, while effective in certain cases (Figure 8c,d at 1150 °C), may not be sufficiently robust to describe the full range of interactions between temperature, strain rate, and microstructural evolution. The relatively simple constitutive framework of the KHL model could be a contributing factor to its reduced accuracy in capturing flow stress variations, particularly at extreme deformation conditions where dynamic recovery and recrystallization mechanisms become dominant. However, the model does demonstrate reasonable accuracy for specific combinations of strain rates and temperatures, likely because it considers the interaction between strain and strain rate. This suggests that while the KHL model provides a better approximation than simpler constitutive models, it still lacks the comprehensive adaptability needed to precisely predict the flow behavior of nickel-based superalloy 925 across diverse processing conditions. These findings emphasize the need for further refinements or modifications to improve the model’s predictive capabilities.

**Figure 9:**
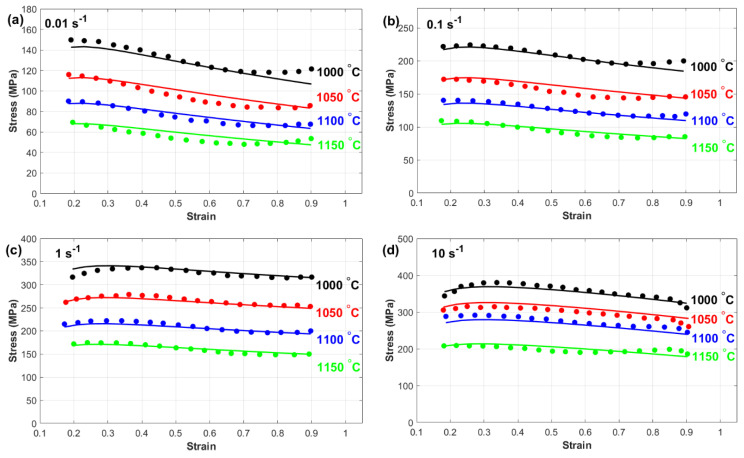
Experimental stress (**markers**) versus predicted stress obtained by the MKD model (**lines**) at (**a**) 0.01 s^−1^, (**b**) 0.1 s^−1^, (**c**) 1 s^−1^, and (**d**) 10 s^−1^.

Figure 9 provides a detailed comparison between the experimentally measured stress and those predicted by the modified Kobayashi–Dodd (MKD) model. The results demonstrate that the MKD model has a high level of accuracy in predicting the flow behavior of the tested nickel-based superalloy across all the examined temperature and strain rate conditions. Unlike its original counterpart, the MKD model successfully aligns with the experimental data, indicating its reliability in capturing the deformation characteristics of a material under varying thermomechanical conditions.

This exceptional predictive performance can be attributed to the model’s ability to effectively incorporate the interactions between strain, strain rate, and temperature. By accounting for these critical factors, the MKD model provides a more comprehensive representation of the alloy’s response to hot deformation. This is particularly important for materials exhibiting complex, nonlinear flow behavior, such as nickel-based superalloy 925, where microstructural changes, including work hardening, dynamic recovery, and recrystallization, play crucial roles in determining stress evolution.

Furthermore, the enhanced accuracy of the MKD model suggests that the modifications made to the original KD model successfully address its limitations, resulting in a more precise constitutive framework. The ability of the MKD model to predict flow stress consistently across a wide range of deformation conditions highlights its potential for optimizing hot-working processes, improving material performance, and extending component service life in high-temperature applications.

**Figure 10:**
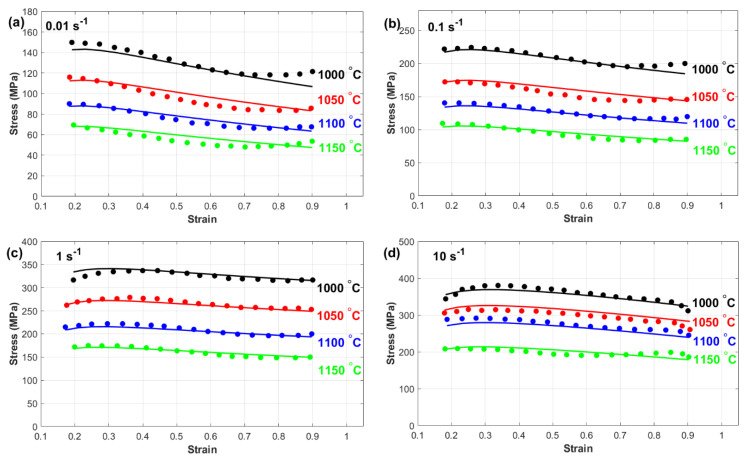
Experimental stress (**markers**) versus predicted stress obtained by the MKHL model (**lines**) at (**a**) 0.01 s^−1^, (**b**) 0.1 s^−1^, (**c**) 1 s^−1^, and (**d**) 10 s^−1^.

Figure 10 presents a comparison between the experimentally measured stress and the predicted stress obtained from the modified Khan–Huang–Liang (MKHL) model. The results clearly demonstrate that the MKHL model accurately represents the flow behavior of nickel-based superalloy 925 across all the examined temperature and strain rate conditions. The close agreement between the experimental and predicted values indicates that the modifications introduced to the original KHL model significantly enhance its predictive ability. This remarkable accuracy can be attributed to the model’s ability to effectively incorporate the interactions among strain, strain rate, and temperature. By integrating these key deformation factors, the MKHL model successfully captures the alloy’s highly nonlinear and complex flow behavior during hot deformation. This is particularly important for materials, such as nickel-based superalloy 925, where microstructural evolution, including strain hardening, dynamic recovery, and recrystallization, plays a significant role in shaping the material’s stress response under different thermomechanical conditions. Moreover, the improved performance of the MKHL model over its original version highlights the importance of considering interdependent deformation characteristics when developing constitutive models. The ability of the MKHL model to predict flow stress consistently across a wide range of temperatures and strain rates makes it a valuable tool for optimizing hot-working processes, improving material processing efficiency, and ensuring the reliability and durability of components used in high-temperature applications.

### 4.2. Validation of the Models

The predictive performance of the KD, KHL, MKD, and MKHL models is systematically evaluated via well-established statistical metrics, including R, AARE, and RMSE, as detailed in [60]. These parameters serve as key indicators for assessing the accuracy and reliability of each model in capturing the flow behavior of the tested nickel-based superalloy 925 under various strain rates and temperatures. The correlation coefficient (R) measures the strength and direction of the relationship between the experimental and predicted stress values, with higher values indicating better model accuracy. The AARE provides insight into the average deviation between the experimental and predicted stress, expressed as a percentage, where lower values signify improved predictive capability. Moreover, the RMSE quantifies the overall magnitude of prediction errors, with smaller values reflecting a closer fit between the model’s predictions and the experimental data. R, AARE, and RMSE are given by:

(38) $$R=\frac{\sum_{i}^{N}\left(\sigma_{e}-\bar{\sigma_{e}}\right)\left(\sigma_{P}-\bar{\sigma_{P}}\right)}{\sqrt{\sum_{i}^{N}{\left(\sigma_{e}-\bar{\sigma_{e}}\right)}^{2}\sum_{i}^{N}{\left(\sigma_{P}-\bar{\sigma_{P}}\right)}^{2}}}$$

(39) $$AARE=\frac{1}{N}\Sigma_{i}^{N}\left|\frac{\sigma_{e}-\sigma_{P}}{\sigma_{e}}\right|\times 100$$

(40) $$RMSE=\frac{1}{N}\sqrt{\Sigma_{i}^{N}}{\left(\sigma_{e}-\sigma_{P}\right)}^{2}$$

**Figure 11:**
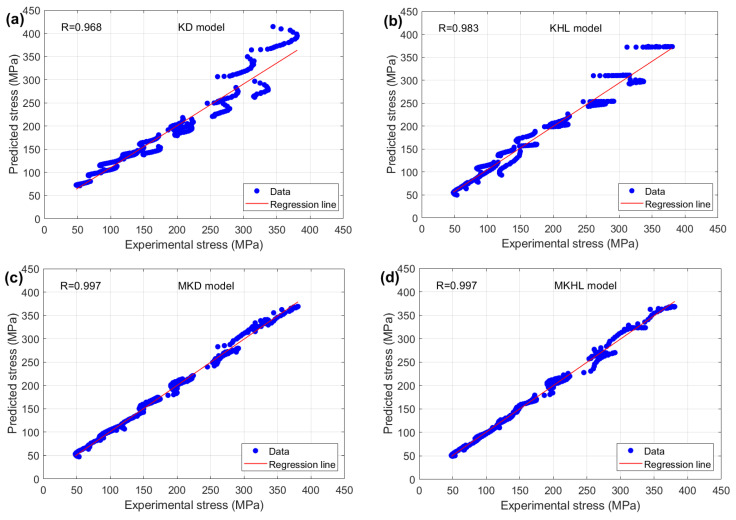
Correlation between experimental stress and predicted stress obtained via (**a**) the KD model, (**b**) the KHL model, (**c**) the MKD model, and (**d**) the MKHL model.

In this context, $\sigma_{e}$ denotes the experimental stress, whereas $\bar{\sigma_{e}}$ signifies the mean value of these experimental stress. Additionally, $\sigma_{P}$ represents the predicted stress, with $\bar{\sigma_{P}}$ indicating the mean value of the predicted stress. Finally, N refers to the number of observations. The predicted stress values obtained from the KD, KHL, MKD, and MKHL models, which are based on Equation (38), are systematically compared with the experimentally measured stress and are visually represented in Figure 11. This comparison provides a clear evaluation of the accuracy and reliability of each model in capturing the flow behavior of the tested nickel-based superalloy 925 under different temperatures and strain rates.

The results presented in Figure 11 indicate that the modified models, MKD and MKHL, exhibit the highest predictive accuracy, achieving an R-value of 0.997 (Figure 11c,d), which signifies an exceptionally strong correlation between the predicted and experimental stress values. In contrast, the original models, KD and KHL, yield relatively low R values of 0.968 and 0.983 (Figure 11a,b). These findings highlight the enhanced performance of the modified models, emphasizing their ability to more accurately capture the complex, nonlinear interactions between strain, strain rate, and temperature during hot deformation. The higher R values observed in the MKD and MKHL models underscore the effectiveness of the modifications introduced, which significantly improves the models’ ability to account for key deformation mechanisms, such as strain hardening, dynamic recovery, and recrystallization. Consequently, these enhanced models provide a more precise and reliable framework for predicting the flow behavior of nickel-based superalloys, making them valuable tools for optimizing hot-working processes and improving material performance in high-temperature applications.

**Figure 12:**
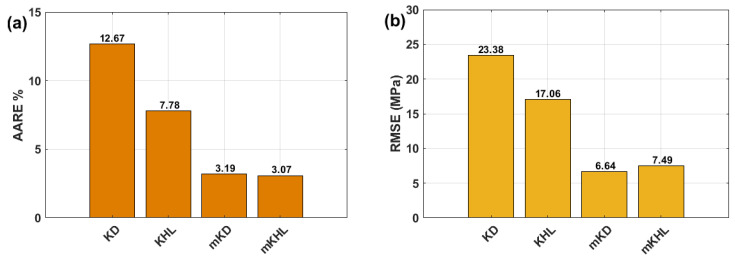
Bar charts for (**a**) AARE (%) and (**b**) RMSE (MPa) obtained by the KD, KHL, MKD, and MKHL models.

The accuracy of the KD, KHL, MKD, and MKHL models in predicting the flow behavior of the tested nickel-based superalloy 925 is further assessed via AARE (see Equation (39)) and RMSE (see Equation (40)). The values obtained for these parameters are visually represented in Figure 12, providing a comprehensive comparison of the predictive performance of each model. The analysis reveals that the modified models, MKD and MKHL, exhibit the lowest AARE values, with 3.19% and 3.07% (Figure 12a), respectively. These significantly lower error percentages indicate that the MKD and MKHL models produce highly accurate predictions that closely match the experimental stress values. In contrast, the original models, KD and KHL, yield substantially higher AARE values of 12.67% and 7.78% (Figure 12a), respectively. These findings suggest that the original models are less effective at capturing the complex deformation behavior of a material, particularly under varying strain rates and temperature conditions.

A similar trend is observed when the RMSE values are evaluated, quantifying the overall deviation between the predicted and experimental stress values. Once again, the MKD and MKHL models outperform the KD and KHL models, achieving the lowest RMSE values of 6.64 MPa and 7.49 MPa (Figure 12b), respectively. Conversely, the original KD and KHL models exhibit notably higher RMSE values of 23.38 MPa and 17.06 MPa (Figure 12b), respectively. These results further confirm that the modifications introduced to the original models significantly enhance their predictive capability.

Overall, the superior performance of the MKD and MKHL models, as evidenced by their lower AARE and RMSE values, underscores the importance of incorporating strain, strain rate, and temperature interactions into constitutive modeling. By effectively capturing an alloy’s complex nonlinear behavior during hot deformation, these modified models offer a more accurate and reliable framework for predicting flow stress, making them valuable tools for optimizing thermomechanical processing and improving material performance in high-temperature applications.

## 5. Conclusions

This study demonstrates that the original Kobayashi–Dodd (KD) and Khan–Huang–Liang (KHL) phenomenological models exhibit inherent limitations in capturing the complex flow behavior of nickel-based superalloy 925 during hot deformation, primarily due to their insufficient ability to account for the coupled effects of strain, strain rate, and temperature. The proposed MKD and MKHL models address these shortcomings through systematic modifications, achieving superior predictive accuracy, as evidenced by statistical metrics (R ≈ 0.997, AARE < 3.2%, RMSE < 7.5 MPa).

The key advantage lies in the ability of the modified models to reconcile the nonlinear interactions between deformation parameters, which is critical for high-temperature applications of nickel-based superalloys. These improvements highlight the importance of incorporating strain-coupled effects into constitutive models for materials exhibiting dynamic recovery and work hardening. For industrial practice, the MKD and MKHL models offer a more reliable framework for optimizing hot-working processes, such as forging or rolling, where precise flow stress predictions are essential to avoid defects and ensure mechanical performance.

Future work should explore the integration of these models into numerical simulations to validate their robustness under broader processing conditions. Additionally, the methodology employed here, combining empirical refinement with physical insights, could be extended to other alloys with similar deformation complexities.

## Figures and Tables

**Table 1:** Constants of the KD model for superalloy 925.

	*σ*_0_ [MPa]	*n*	*m*	*β*
Superalloy 925	259.86	−0.081	0.143	0.0032

**Table 2:** Constants of the KHL model for superalloy 925.

	*A* [MPa]	*B* [MPa]	*n*_1_	*n*_0_	*C*	*m*
Superalloy 925	316.50	−25.64	2.899	0.322	0.075	0.933

**Table 3:** Constants of the MKD model for superalloy 925.

	*σ*_0_ [MPa]	*n*_0_	*n*_1_	*m*_0_	*m*_1_	*m*_2_
Superalloy 925	311.61	−0.137	0.018	0.171	0.072	−0.015
	*m*_3_	*β*_0_	*β*_1_	*β*_2_	*β*_3_	*β*_4_
	−0.0009	0.0045	0.0003	−0.0002	−7.19 × 10^−6^	5.94 × 10^−10^

**Table 4:** Constants of the MKHL model for superalloy 925.

	*A* [MPa]	*B* [MPa]	*n*_10_	*n*_11_	*n*_12_
Superalloy 925	316.50	6.54	12.004	0.417	0.225
	*n*_13_	*C*_0_	*C*_1_	*C*_2_	*C*_3_
	−0.244	0.349	0.140	−0.134	−0.035
	*m*_0_	*m*_1_	*m*_2_	*m*_3_	*m*_4_
	1.023	−0.198	0.063	−0.141	0.032

## Data Availability

Data will be available upon request through the corresponding authors.
